# Tuning In-Plane Magnetic Anisotropy and Interfacial Exchange Coupling in Epitaxial La_2/3_Sr_1/3_CoO_3_/La_2/3_Sr_1/3_MnO_3_ Heterostructures

**DOI:** 10.1021/acsami.3c10376

**Published:** 2023-11-01

**Authors:** Mingzhen Feng, Nolan Ahlm, Dayne Y. Sasaki, I-Ting Chiu, Alpha T. N’Diaye, Padraic Shafer, Christoph Klewe, Apurva Mehta, Yayoi Takamura

**Affiliations:** † Department of Materials Science and Engineering, University of California, Davis, Davis, California 95616, United States; ‡ Department of Chemical Engineering, University of California, Davis, Davis, California 95616, United States; § Advanced Light Source, Lawrence Berkeley National Laboratory, Berkeley, California 94720, United States; ∥ Stanford Synchrotron Radiation Lightsource, SLAC National Accelerator Laboratory, Menlo Park, California 94025, United States

**Keywords:** X-ray magnetic circular dichroism, magnetic anisotropy, exchange bias, perovskite oxides, interface, X-ray linear dichroism

## Abstract

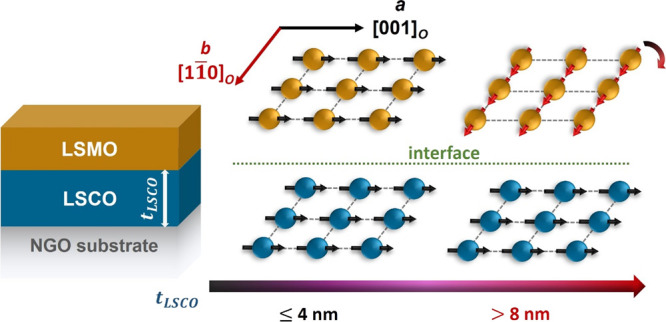

Controlling the in-plane magnetocrystalline anisotropy and interfacial exchange coupling between ferromagnetic (FM) layers plays a key role in next-generation spintronic and magnetic memory devices. In this work, we explored the effect of tuning the magnetocrystalline anisotropy of La_2/3_Sr_1/3_CoO_3_ (LSCO) and La_2/3_Sr_1/3_MnO_3_ (LSMO) layers and the corresponding effect on interfacial exchange coupling by adjusting the thickness of the LSCO layer (*t*_LSCO_). The epitaxial LSCO/LSMO bilayers were grown on (110)_*o*_-oriented NdGaO_3_ (NGO) substrates with a fixed LSMO (top layer) thickness of 6 nm and LSCO (bottom layer) thicknesses varying from 1 to 10 nm. Despite the small difference (∼0.2%) in lattice mismatch between the two in-plane directions, [001]_*o*_ and [11̅0]_*o*_, a pronounced in-plane magnetic anisotropy was observed. Soft X-ray magnetic circular dichroism hysteresis loops revealed that for *t*_LSCO_ ≤ 4 nm, the easy axes for both LSCO and LSMO layers were along the [001]_*o*_ direction, and the LSCO layer was characterized by magnetically active Co^2+^ ions that strongly coupled to the LSMO layer. No exchange bias effect was observed in the hysteresis loops. In contrast, along the [11̅0]_*o*_ direction, the LSCO and LSMO layers displayed a small difference in their coercivity values, and a small exchange bias shift was observed. As *t*_LSCO_ increased above 4 nm, the easy axis for the LSCO layer remained along the [100]_*o*_ direction, but it gradually rotated to the [11̅0]_*o*_ direction for the LSMO layer, resulting in a large negative exchange bias shift. Therefore, we provide a way to control the magnetocrystalline anisotropy and exchange bias by tuning the interfacial exchange coupling between the two FM layers.

## 1 Introduction

Exchange bias (EB) is an effect broadly explored in ferromagnetic (FM)/antiferromagnetic (AFM) heterostructures^1−5^ due to its promising applications in devices such as permanent magnets,^6,7^ spin valves,^8,9^ and magnetic recording read heads.^10,11^ It is characterized by a horizontal shift of magnetic hysteresis loops in the direction opposite to (negative EB)^12−14^ or along with (positive EB)^15−18^ the biasing or field cooling direction. While it has been widely studied on FM metals and alloys, magnetic perovskite oxides provide degrees of tunability which are absent in purely metallic materials due to interactions between the charge, orbital, spin, and lattice degrees of freedom.^19−22^ EB in perovskite oxides has not only been found in FM/AFM heterostructures but has also been observed at interfaces between hard and soft FM layers.^19,20,23,24^

Tuning the magnetocrystalline anisotropy (MA) of different layers in a heterostructure offers the potential to influence the EB effect. Among the perovskite oxides, La_2/3_Sr_1/3_MnO_3_ (LSMO) has been extensively studied due to the ability to control its MA properties.^25−27^ For example, Liao et al. demonstrated the switch from interfacial magnetic anisotropy (IMA) to bulk magnetic anisotropy (BMA) of the magnetic easy axis in ultrathin LSMO layers grown on (110)_*o*_-oriented NdGaO_3_ (NGO) substrates by the insertion of an SrTiO_3_ (STO) buffer layer.^27^ Chen et al. further demonstrated that MA in the LSMO layer could be probed through the symmetry of the Mn 3*d* orbitals, where the 3*d*_*x*^2^–*y*^2^_ occupancy showed a direct relationship with in-plane anisotropy.^26^ However, the EB effect could not be explored on the LSMO/STO system due to the diamagnetic properties of the STO layers. Although it has been reported that the magnetic easy axis of La_0.8_Sr_0.2_CoO_3_/LSMO bilayer can be rotated from the out-of-plane to in-plane (IP) direction via ionic-liquid gating, thus simultaneously reducing and reversing the exchange bias,^28^ it is not clear which layer contributes to the EB effect from bulk magnetometry. In this study, we present a new approach of tuning the *bulk-like* LSMO (∼6 nm) easy axis from BMA to IMA by changing the thickness of the underlying La_2/3_Sr_1/3_CoO_3_ (LSCO) layer in LSCO/LSMO bilayers deposited on (110)_o_-oriented NGO substrates and utilizing soft X-ray magnetic circular dichroism (XMCD) hysteresis loops to gain insight of the properties of each layer. This approach allows for enhanced control over the EB behavior of the LSMO layer.

In prior studies, we reported on the exchange coupling in LSCO/LSMO bilayers grown on (LaAlO_3_)_0.3_(Sr_2_AlTaO_6_)_0.7_ (LSAT) substrates and demonstrated that exchange spring behavior was observed where the hard LSCO layer (h-LSCO) biased a composite soft layer.^19,20^ This composite soft layer consisted of an interfacial soft LSCO layer (s-LSCO) characterized by magnetically active Co^2+^ ions and an LSMO layer. This s-LSCO layer arose due to the formation of oxygen vacancies and interfacial charge transfer,^24^ and as a result, the EB effect was only found when the LSCO thickness (*t*_LSCO_) exceeded a critical thickness. Unlike the cubic-structured LSAT substrates where the substrate/film lattice mismatch is equal along the two orthogonal in-plane directions, a small difference (∼0.2%) of lattice mismatch between the two in-plane directions exists in (110)_*o*_-NGO substrates.^29^ In a recent study, LSCO/LSMO bilayers on NGO substrates showed a similar Co ion distribution throughout the LSCO thickness: a nonmagnetic layer characterized by Co^3+^ ions at the LSCO/NGO interface, a bulk-like h-LSCO layer with mixed Co^3+^/Co^4+^ ions in the middle of the layer, and an FM s-LSCO layer at the LSCO/LSMO interface.^30^ Moreover, the formation of the Co^3+^/Co^4+^ ions was suppressed as *t*_LSCO_ decreased, leaving only an s-LSCO layer with strong ferromagnetism. However, the interfacial exchange coupling behavior and MA for the bilayers on NGO substrates were not explored.

In this work, epitaxial LSCO/LSMO bilayers were grown on (110)_*o*_-oriented NGO substrates with a fixed LSMO thickness of 6 nm, and LSCO thicknesses varied from 1 to 10 nm. NGO has orthorhombic (o) symmetry with the *a*^*–*^*a*^*–*^*c*^*+*^ tilt pattern in the Glazer notation^31^ and can be redefined as a pseudocubic (pc) unit cell with a slightly rectangular in-plane lattice (*a*_pc_//[001]_*o*_ = 3.855 Å, *b*_pc_//[11̅0]_*o*_ = 3.863 Å, and *c*_pc_//[110]_*o*_ = 3.855 Å).^29^ The LSCO layer exists under in-plane tensile strain (0.57% along the ***a***-direction, 0.78% along the ***b***-direction) on NGO substrates, while the LSMO layer is under in-plane compressive strain (0.54% along the ***a****-*direction, 0.34% along the ***b****-*direction) on NGO substrates where the in-plane strain is defined as ε = *(a*_film –_*a*_substrate_*)/a*_substrate_. The BMA of LSCO and LSMO single layers on (110)_*o*_-NGO substrates is along the ***a-*** ([001]_*o*_) and ***b-*** ([11̅0]_*o*_) directions, respectively, corresponding with the smaller in-plane strain induced by the substrate.^29^ Based on the results of XMCD hysteresis loops taken at the Co and Mn *L*-edges, we found that the easy axes for the LSCO layers were always aligned along the ***a***-direction regardless of *t*_LSCO_. Surprisingly, the LSMO sublayer clearly saw a transition from an easy axis along the ***a***-direction for *t*_LSCO_ ≤ 4 nm, to the ***b***-direction for larger *t*_LSCO_ values, despite the fact that the LSMO sublayer thickness remains fixed at 6 nm, suggesting the influence of a unique IMA that differs from the bulk properties. As a consequence, for *t*_LSCO_ ≤ 4 nm, no EB effect was observed along the ***a***-direction, while a small negative EB shift was observed along the ***b***-direction. For *t*_LSCO_ > 4 nm, a large negative EB shift was found along both ***a-*** and ***b-***directions due to the differing in-plane MA between the LSCO and LSMO layers. The preferred direction of the Mn 3*d*_*x*^2^ – *y*^2^_ orbital occupancy was measured by element-specific X-ray linear dichroism (XLD) and correlated to the in-plane MA of the LSMO layer. Therefore, the interfacial coupling between the LSCO and LSMO layers strongly influences the IMA of the LSMO layer, enabling the control of EB between the two FM layers.

## 2 Experimental Methods

### 2.1 Thin-Film Deposition

The LSCO/LSMO bilayers were grown on (110)_*O*_-oriented NGO substrates by pulsed laser deposition from stoichiometric La_2/3_Sr_1/3_CoO_3_ and La_2/3_Sr_1/3_MnO_3_ targets. The LSCO layer thickness was designed to range from 1 to 10 nm, capped with 6 nm of LSMO, referred to as bilayers CxM6N (*x* = 1–10, corresponding to the LSCO layer thickness in nanometer). The chamber was pumped to a base pressure of 2 × 10^–6^ Torr then subsequently filled with flowing O_2_ gas to a fixed pressure of 300 mTorr. During the deposition, the substrate temperature was held at 700 °C and a KrF excimer laser (λ = 248 nm), with 1.0 J/cm^2^ laser energy and 1 Hz laser repetition rate was used for both LSCO and LSMO layers. The bilayers were cooled to room temperature in 300 Torr O_2_ at a rate of 10 °C/min to ensure proper oxygen stoichiometry.

### 2.2 Structural Property Characterization

The structural properties of the bilayers (e.g., total film thickness, interface roughness, crystallinity, and strain state) were characterized by X-ray reflectivity (XRR), high-resolution X-ray diffraction (XRD), and reciprocal space maps (RSMs) using a Bruker D8 Discover four-circle X-ray diffractometer using Cu K_α1_ X-rays (λ = 1.5406 Å). The out-of-plane lattice parameters of the bilayers were obtained by fitting the XRD profiles using Leptos software.^32^ Due to the similarities of the densities of LSCO and LSMO, the bilayers were further characterized using resonant XRR (RXRR) measurements at Beamline 2–1 of the Stanford Synchrotron Radiation Lightsource (SSRL). RXRR profiles were measured at the Co *K*-edge (7730 eV), Mn *K*-edge (6558 eV), and an off-resonance energy (8000 eV). The higher sensitivity of RXRR measurements was able to probe the structural properties of each layer in the bilayers.^33^ By fitting the RXRR profiles at three energies to one structural model using GenX software,^34^ the thickness, roughness, and density of each layer were determined.

### 2.3 Magnetic Properties

Soft X-ray magnetic spectra (X-ray absorption (XA), XMCD, and XLD spectra) were acquired at the Co and Mn *L*-edges at 80 K using Beamline 4.0.2 of the Advanced Light Source (ALS) in total electron yield (TEY) mode, which probes the top 5–10 nm of the bilayer (corresponding to the entire LSMO layer and LSCO/LSMO interface region), limited by the escape length of secondary electrons.^35^ For the acquisition of the XMCD spectra, the bilayers were field cooled to 0.3 T to ensure that all magnetic moments are aligned along the field direction. During the measurements, a 0.3 T magnetic field was applied parallel to the incident X-ray beam which was 60° from the surface normal, and XA spectra were collected using right- and left-circularly polarized X-rays. XMCD spectra were calculated as the difference between two jointly normalized XA spectra collected with right (*I*_R_) and left (*I*_L_) circularly polarized X-rays. The XLD spectra were acquired with the X-ray beam perpendicular to the sample surface, and the direction of the linear polarization vector was oriented with *E⃗*//***a*** and *E⃗*//***b***. The XLD spectra were calculated as the difference of the XA spectra obtained with the two linear polarization directions. Bilayers were zero-field cooled to 80 K before all XMCD hysteresis loop measurements. Unbiased Mn-XMCD hysteresis loops (Figure 2a–d) and unbiased Co-XMCD hysteresis loops for bilayer C1M6N (Figure 2e) were measured in TEY mode at Beamline 4.0.2 of the ALS with the magnetic field applied along the ***a-*** and ***b-***directions. Unbiased Co-XMCD hysteresis loops for thicker bilayers (*t*_LSCO_ ≥ 4 nm, Figure 2f–h) and biased Mn-XMCD hysteresis loops were measured at 80 K in TEY mode along the ***a-*** and ***b****-*directions using Beamline 6.3.1 of the ALS. For the biased hysteresis loops, the bias field was set to ±1.8 T for 1 min then the minor loops were measured from −0.3 T to +0.3 T.

## 3 Results and Discussion

**Figure 1:**
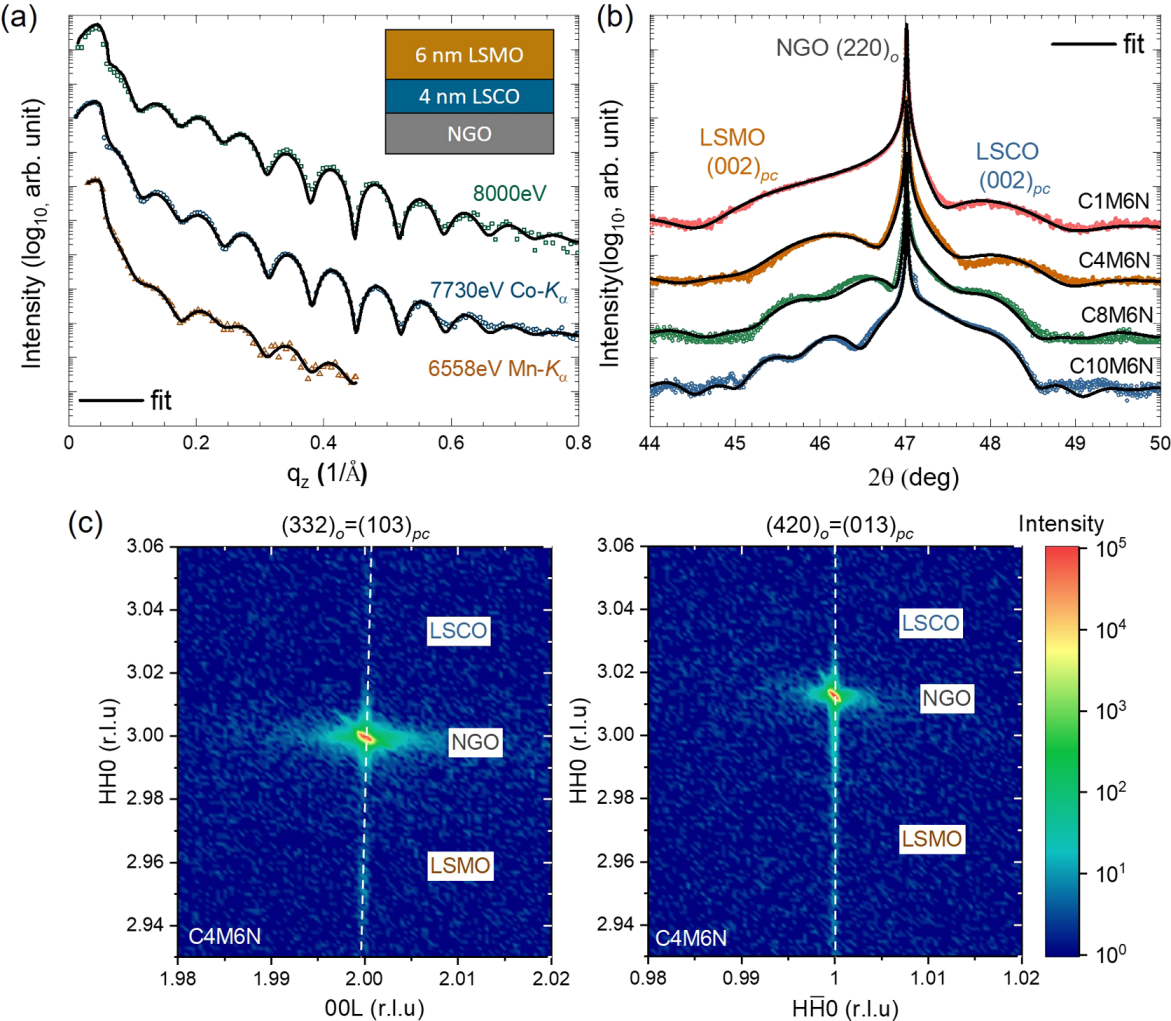
(a) RXRR spectra for bilayer C4M6N and (b) XRD 2θ scans of C*x*M6N (*x* = 1, 4, 6, 10) bilayers. Colored symbols are experimental data, and black curves are fitting results corresponding to the parameters listed in Table SI. Curves are vertically shifted for clarity. (c) RSMs for bilayer C4M6N around the (103)_*pc*_ and (013)_*pc*_ reflections. The dashed white line marks the in-plane alignment of the film and substrate peaks.

The LSCO/LSMO bilayers were characterized by RXRR and the curves were fit using GenX software^34^ to determine the individual layer thickness, roughness, and density parameters. RXRR curves for bilayer C4M6N are shown in Figure 1a as an example where all three energy spectra were fit simultaneously to one structural model, and the parameters derived from the best fits are listed in Table SI. A thin carbon capping layer was added to the fitting model due to the extended exposure to hard X-rays in air during the measurements. Kiessig fringes can be seen in all curves, indicating smooth surface/interface regions. The density of each layer at the interface region is lower than the bulk values as listed in Table SI, suggesting the formation of oxygen vacancies at the interface.^24,36,37^ The total thickness of each layer is in a good agreement with expectations and the fitting results agree with our previous studies on LSCO/LSMO bilayers.^23,24^

Figure 1b shows XRD ω–2θ scans of the LSCO/LSMO bilayers around the (220)_*o*_ reflection of the NGO substrates. Curves are vertically shifted with *t*_LSCO_ values ranging from 1 to 10 nm. The LSMO and LSCO (002)_*pc*_ film peaks are on the left and right sides of the NGO substrate peak, respectively, due to the different strain states between the film and the substrate. Though it is difficult to denote film positions because of the overlap between the two film peaks and the substrate peak, the scans were fitted using Leptos software^32^ to obtain out-of-plane lattice parameter (*c*) and *c/a* ratio of each layer. Fit curves are plotted in black, and the fitting results are listed in Table 1.

**Table 1:** XRD Curve Fit Parameters for CxM6N Bilayers (*x* = 1, 4, 8, 10)

bilayer CxM6N	*c*_LSMO_ (Å)	*c*_LSMO_*/a*	*c*_LSCO_ (A°)	*c*_LSCO_*/a*
*x* = 1	3.911	1.012	3.843	0.995
*x* = 4	3.910	1.012	3.824	0.990
*x* = 8	3.906	1.011	3.829	0.991
*x* = 10	3.909	1.012	3.811	0.987

*c*_LSMO_*/a* values for the LSMO layer are almost the same regardless of *t*_LSCO_ but slightly larger than the values reported for a single-layer LSMO film on NGO substrates,^38,39^ suggesting that the underlying LSCO layer may affect the interfacial octahedral tilt pattern/angles of the LSMO layer on top. For the LSCO layer, an overall trend of increased *c*_LSCO_ is observed as *t*_LSCO_ decreased, which can be caused by a higher concentration of oxygen vacancies and Co^2+^ ions at the LSCO/LSMO interface^19,24^ which have a larger radius than Co^3+^ and Co^4+^ ions (high-spin octahedral coordinated Co^2+^: 88.5 pm, low-spin octahedral coordinated Co^3+^: 68.5 pm, high-spin octahedral coordinated Co^4+^: 67 pm).^40,41^ The detailed information regarding Co ion spin and valence states is discussed later in this article. RSMs were measured around the (103)_*pc*_ and (013)_*pc*_ reflections which are rotated by 90° from one another to probe the structural information along the two inequivalent in-plane directions of the orthorhombic NGO substrate. In Figure 1c, RSMs of bilayer C4M6N are shown as an example, where vertical alignment of film peaks with substrate peak indicates that both layers are coherently strained to the NGO substrate. Therefore, no strain relaxation was found for bilayers with *t*_LSCO_ up to 10 nm.^30^ In addition, a small tilt is observed between the film and substrate peak locations in the (103)_*pc*_ RSM (marked as the white dashed line), indicating that the film has a unit cell angle deviation away from 90° along this direction. This deviation is only observed in the (103)_*pc*_ RSM meaning that this angle variation is only occurring for one of the unit cell angles, resulting in monoclinic unit cells for the LSCO and LSMO films on NGO substrates. The observed unit cell angle deviation has also been reported for other perovskite films on orthorhombic substrates, such as SrRuO_3_ films on DyScO_3_ and GdScO_3_ substrates.^42,43^ Thus, the small tilt in the RSM suggests BO_6_ octahedral reconstruction at the heterostructure interfacial.^44,45^

**Figure 2:**
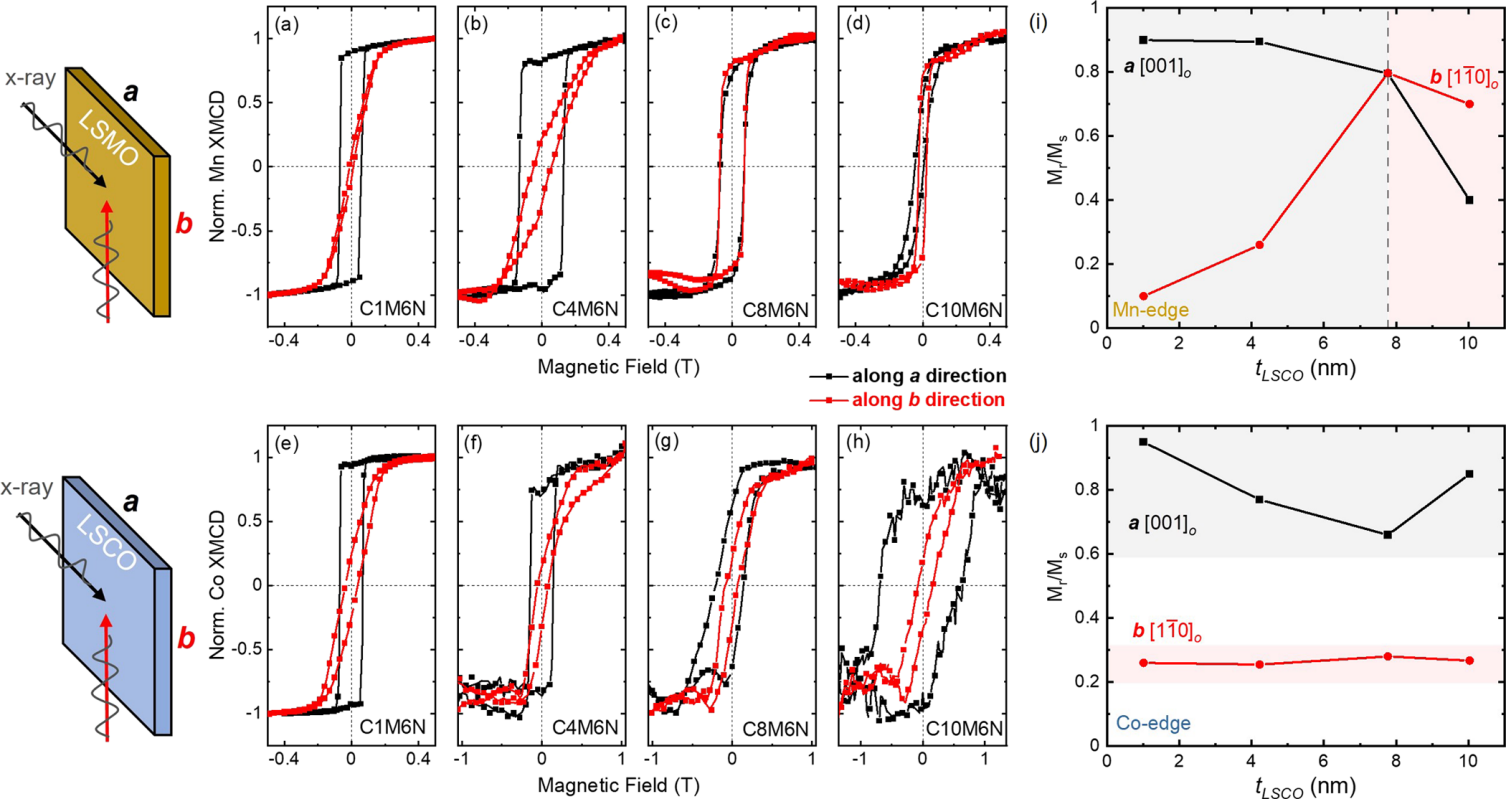
Unbiased XMCD loops acquired at the Mn *L*-edge (a–d) and at the Co *L*-edge (e–h) measured along the ***a**-* (black) and ***b**-* (red) directions. Loops were normalized from −1 to 1. Squareness (*M*_r_*/M*_s_) of XMCD loops along ***a-*** and ***b-***directions at the Mn-edge (i) and at the Co-edge (j) are plotted as a function of LSCO layer thickness (*t*_LSCO_).

**Figure 3:**
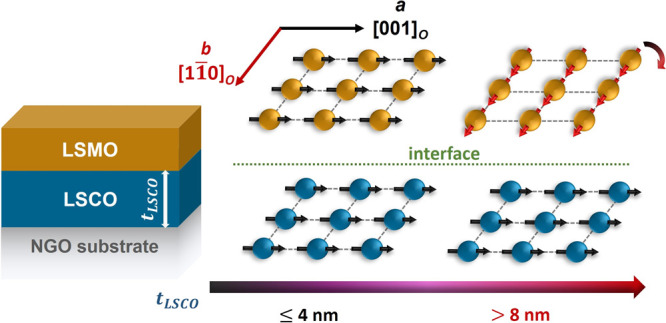
LSCO thickness (*t*_LSCO_)-dependent in-plane MA in LSCO/LSMO bilayers on NGO substrates. The Mn ions are colored in yellow and Co ions are colored in blue.

To explore the in-plane MA of the LSMO and LSCO layers as a function of *t*_LSCO_, unbiased XMCD loops at the Mn and Co *L*-edges were acquired in TEY mode immediately after zero field cooling to 80 K. Traditional bulk magnetometry cannot separate the magnetic contributions of individual layers; however, by tuning the X-ray energies to either the Co or Mn *L*-edges enables such element-specificity from XMCD measurements. Figure 2a–d plots the unbiased Mn-XMCD minor loops measured along *a***-** and ***b-***directions with increasing *t*_LSCO_, and they provide information on the inherent LSMO magnetic anisotropy while the LSCO layer is in the demagnetized state. The squareness of these loops, defined as remanent magnetization (*M*_r_)/saturation magnetization (*M*_s_) is plotted in Figure 2i. The squareness values are much higher along the ***a-***direction (about 0.9) than along the ***b-***direction when *t*_LSCO_ < 8 nm, indicating the magnetic easy axis of LSMO layer is along the ***a*** ([001]_*o*_) direction. However, the in-plane MA of the LSMO layer switches to the ***b*** ([11̅0]_*o*_) direction when *t*_LSCO_ increases to 10 nm. For *t*_LSCO_ = 8 nm, the loop shape and squareness along ***a*****-** and ***b-***directions are almost equal, suggesting that either the magnetic easy axis lies between the ***a-*** and ***b-***directions or both in-plane directions are energetically degenerate. The trend of *M*_r_*/M*_S_ vs *t*_LSCO_ along the two in-plane directions reveals that the magnetic properties of the LSMO layer switch from IMA to BMA at a critical *t*_LSCO_ of 8 nm. The *t*_LSCO_-dependent in-plane MA of the LSMO layer is shown schematically in Figure 3. It should be noted that the easy axis of bulk LSMO single layers on (110)_*o*_-NGO substrate is along the ***b-***direction, due to slightly smaller in-plane strain.^29^ The reorientation of the LSMO easy axis to the ***a-***direction reveals that the strain state is no longer the dominant effect in determining the LSMO MA. We propose that the interfacial interactions between LSCO and LSMO layers plays a key role in explaining this behavior.

Similar unbiased XMCD loops at the Co *L*-edge were taken along the ***a***- and ***b**-*directions as shown in Figure 2e–h. Note that the magnetic field range is significantly expanded for the three thicker bilayers. The squareness values of the Co-edge loops along the ***a-***direction are much larger than those along the ***b**-*direction regardless of *t*_LSCO_ as shown in Figure 2j, suggesting that the easy axis of the LSCO layer is always along the ***a*** ([001]_*o*_) direction, which is in agreement with single layer LSCO on NGO substrates. In addition, a trend of increasing *H*_c_ (LSCO) as a function of *t*_LSCO_ is observed (as shown in Figure S1), similar to the behavior of single-layer LSCO on NGO and other substrates.^30,46^ Furthermore, in bilayers C1M6N and C4M6N, *H*_c_ (LSCO) is almost the same as *H*_c_ (LSMO) along the ***a-***direction (as shown in Figure S1(a)), indicating that the LSCO and LSMO layers are magnetically coupled when *t*_*LSCO*_ ≤ 4 nm. It has been reported that LSCO and LSMO layers were fully magnetically coupled when *t*_LSCO_ was below a critical thickness, as indicated by a single magnetic switching event from bulk magnetometry.^19,30^ This interfacial coupling results in an increase of *H*_*c*_ (LSMO) values as shown in Figure S1 compared to that of single-layer LSMO on NGO and other substrates. Along the ***a-***direction, *H*_c_ (LSMO) = 0.13 T in bilayer C4M6N, which corresponds to an enhancement of more than 60 times when compared to the bulk value (∼0.002 T)^47,48^ and more than six times compared to the thickest bilayer C10M6N (∼0.02 T). *H*_c_ enhancement is commonly observed in FM/AFM systems due to the pinning effect^49,50^ or caused by the presence of defects in the FM sample^48^ but has not been widely studied in FM/FM perovskite systems. Therefore, the modification of *H*_c_ values and change in the LSMO MA through interfacial coupling could affect the EB effect in LSCO/LSMO heterostructures.

**Figure 4:**
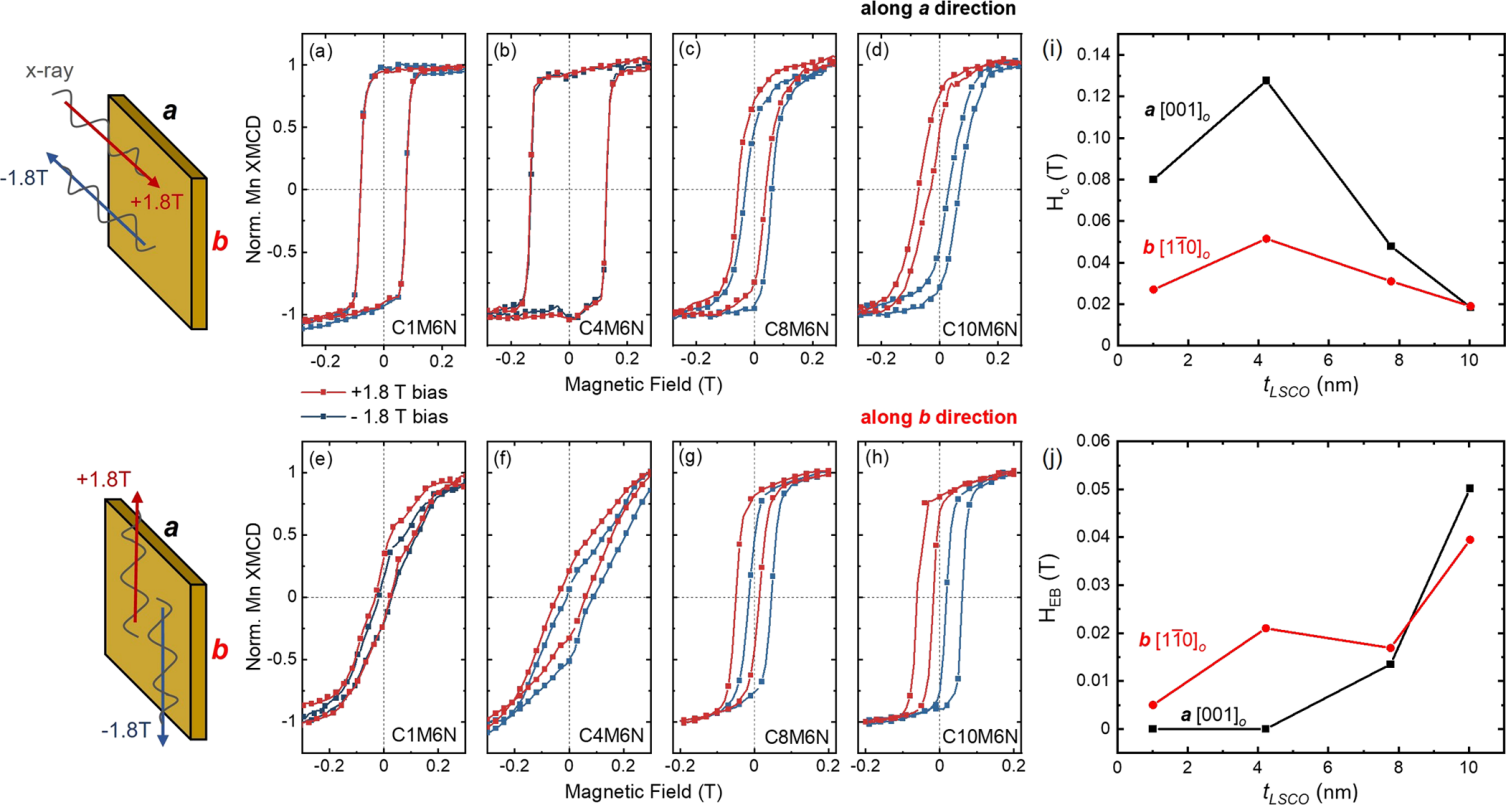
Biased XMCD minor loops acquired at the Mn *L*-edge measured along the ***a*****-**direction (a–d) and along the ***b*****-**direction (e–h). Loops in red and blue were measured after applying a +1.8 T field and a −1.8 T field, respectively. Loops were normalized from −1 to 1. *H*_*c*_ values of biased loops in (i) and EB field (*H*_EB_) in (j) as a function of LSCO layer thickness (*t*_LSCO_) along the ***a**-* and ***b**-*directions.

The exchange bias effect of the LSCO/LSMO bilayers is explored using biased XMCD hysteresis loops measured in TEY mode along the ***a**-* and ***b****-*directions after zero-field cooling to 80 K as shown in Figure 4. Unlike in traditional FM/AFM exchanged bias systems, biasing in FM/FM systems does not require field-cooling the samples through the Néel temperature of the AFM layer but can be accomplished by magnetizing both hard and soft layers with a sufficiently large magnetic field (i.e., 1.8 T) or field cooling the sample through the Curie temperature of both layers. At the Mn *L*-edge, the bilayers were first biased at +1.8 T or −1.8 T so that both layers reach full saturation, then minor loops were measured between ±0.3 T. Figure 4a–d plots biased XMCD minor loops at the Mn *L*-edge measured along the ***a*****-**direction with *t*_LSCO_ ranging from 1 to 10 nm. For *t*_LSCO_ ≤ 4 nm, the absence of an EB effect in bilayers C1M6N and C4M6N along the ***a***-direction, as observed by perfectly overlapping minor loops, can be attributed to the same MA and the magnetic coupling between the hard/soft FM layers. Conversely, a small EB effect is observed along the ***b***-direction in the two thinner bilayers (shown in Figure 4e,f), caused by slightly different *H*_c_ values between the two FM layers (shown in Figure S1(b)). For bilayers with *t*_LSCO_ > 4 nm, a significant lateral shift of the hysteresis loops in the direction opposite to the biasing field is observed along both in-plane directions due to the different in-plane MA and larger difference in *H*_c_ values between the LSCO and LSMO layers, resulting in a large EB effect. Such behavior indicates that the magnetic moments of the soft LSMO layer are pinned by a hard FM layer, and the interfacial FM/FM exchange coupling is similar to the behavior at FM/AFM interfaces.^51,52^ Unfortunately, experimental limitations due to the extremely low luminescence yield of NGO substrates^30^ and the finite probing depth of TEY detection mode^35^ prevent us from acquiring reliable biased Co-XMCD loops from the buried LSCO layer.

The *H*_c_ values from Mn-XMCD minor loops and exchange bias field (*H*_EB_) values as a function of *t*_LSCO_ are plotted in Figure 4i,j, respectively. *H*_EB_ values were found to increase as a function of *t*_LSCO,_ where *H*_EB_ is defined as *H*_EB_ = |(*H*_1_ + *H*_2_)/2|, and *H*_1_ and *H*_2_ are the values where the hysteresis loop intersects the left and right field axes, respectively. In EB systems, the expectation is that *H*_EB_ and *H*_c_ should show similar trends.^53^ However, a reversed trend of *H*_EB_ and *H*_c_ is observed in Figure 4i,j, suggesting that additional interfacial interactions must be considered. A similar decoupling of *H*_EB_ and *H*_c_, and the enhancement of *H*_c_ values in LSCO/LSMO bilayers was previously reported, where the decrease in interfacial Co^2+^ ion concentration and an increase of nonmagnetic Co^3+^/Co^4+^ ions was observed with increasing *t*_LSCO_.^23^ Here we propose a similar *t*_LSCO_ dependence of the hard/soft magnetic interfacial interactions related to the details of the Co valence states in the LSCO layer, as discussed below.

**Figure 5:**
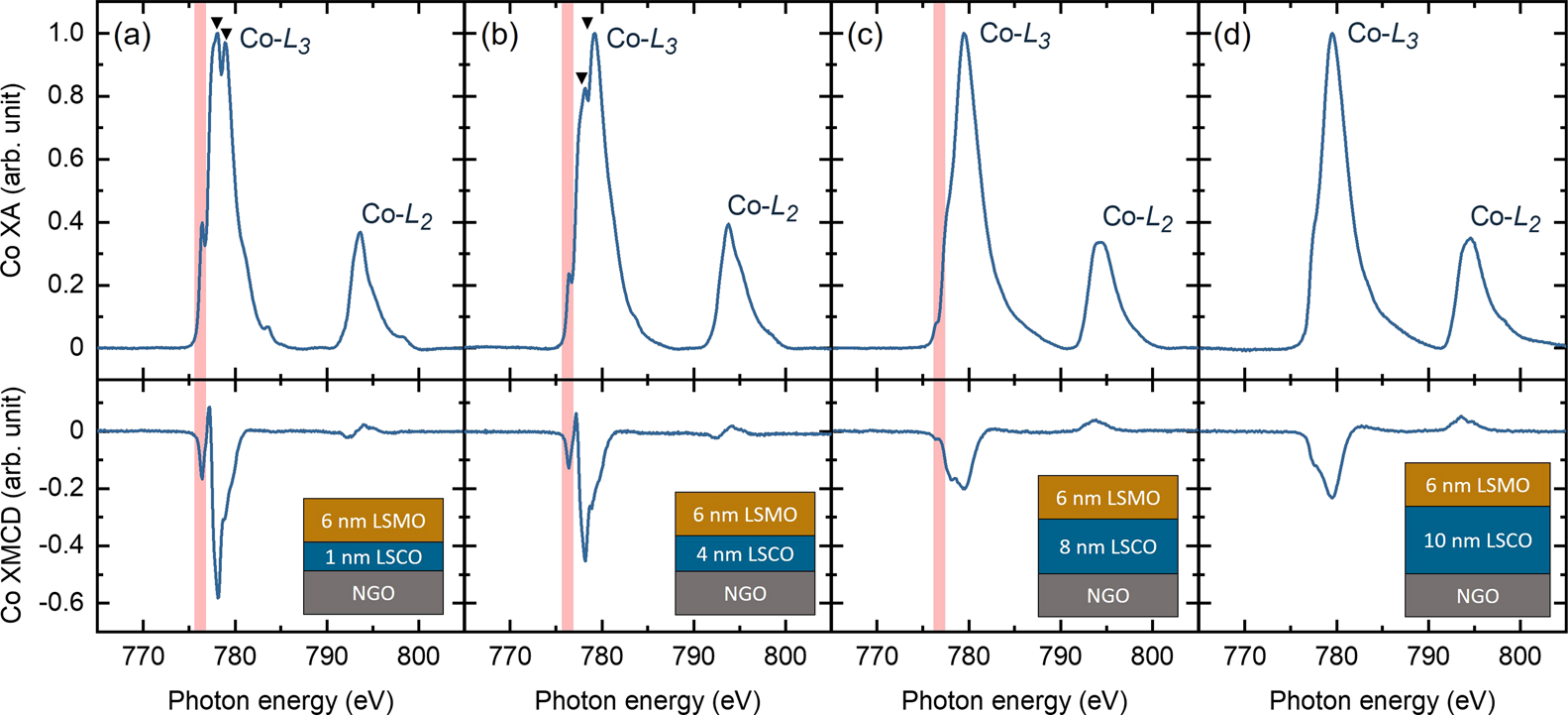
(a–d) Co *L*-edge XA/XMCD spectra of CxM6N (*x* = 1, 4, 8, 10) bilayers taken in the TEY mode (interfacial region). XA spectra are normalized from 0 to 1. Prepeak features are shaded with pink color, while “▼” symbols represent the main *L*_*3*_ peaks associated with Co^2+^ ion features.

Co ion valence states and bonding configurations in the bilayers were probed by Co *L*-edge XA spectra as shown in Figure 5. Averaged XA spectra were normalized from 0 to 1. Reference spectra from CoFe_2_O_4_ (Co^2+^) and a single-layer LSCO film (mixed Co^3+^/Co^4+^), both in octahedral coordination, are plotted in Figure S2 for comparison to the bilayer spectra. The Co-XA spectra of bilayer C1M6N shown in Figure 5a reveal that the Co ions are predominantly Co^2+^ ions. For the thickest bilayer C10M6N, the Co-XA curve fully assembles the single-layer LSCO reference spectra where mixed Co^3+^/Co^4+^ ions dominate as shown in Figure 5d. In addition, several general trends can be observed: (1) as *t*_LSCO_ increases to 8 nm, the prepeak intensity (shaded in pink color) decreases and fully disappears when *t*_LSCO_ = 10 nm; (2) the intensity ratio of the doublet peak (marked with “▼”) reduces as *t*_LSCO_ increases from 1 to 4 nm; and (3) the Co-*L*_3_ peak positions shift to higher energies as *t*_LSCO_ increases. These gradual changes in XA spectral shape indicate that the valence state of Co ions is gradually changing from Co^2+^-dominated to mixed Co^3+^/Co^4+^-dominated with increasing *t*_LSCO_, which is consistent with the results in previous work.^20,24,30^ This *t*_LSCO_ dependence again supports the result of decoupling of *H*_EB_ and *H*_c_, and the enhancement of *H*_c_ values in the LSMO layer as shown in Figure 4.^23^

The Co-XMCD spectra denote magnetically active Co ions in the bilayers. The XMCD signal from bilayers C1M6N and C4M6N where Co^2+^ ions dominate is much stronger than thicker bilayers where mixed Co^3+^/Co^4+^ ions predominate, suggesting that the Co^2+^ ions are in a high spin state.^24,54^ Moreover, the decrease of Co-XMCD intensity as *t*_LSCO_ increases from 1 to 8 nm indicates that the number of Co^2+^ ions does not increase with *t*_LSCO_ but rather is replaced by mixed Co^3+^/Co^4+^ ions that have lower magnetization. The existence of high spin Co^2+^ ions (larger ionic radius) in thinner bilayers is also supported by the observation of *c*-lattice expansion shown in Table 1. The formation of Co^2+^ ions cannot be explained by the charge transfer between Co and Mn ions due to the fact that the valence state of Mn ions stays almost unchanged with *t*_LSCO_ as shown in Figure S3, thus we propose the contribution from oxygen vacancies at the LSCO/LSMO interface to maintain charge neutrality.^24,30^Figure S4 displays the O *K*-edge spectra from the LSCO/LSMO bilayers, revealing a decreasing trend of peak A (ascribed to transition metal 3*d* unoccupied states) and an increasing intensity of peak B (transition metal 3*d* relevant absorption peak) with increasing LSCO thickness.^55,56^ These observations suggest the presence of more oxygen vacancies in the thinner bilayers.

**Figure 6:**
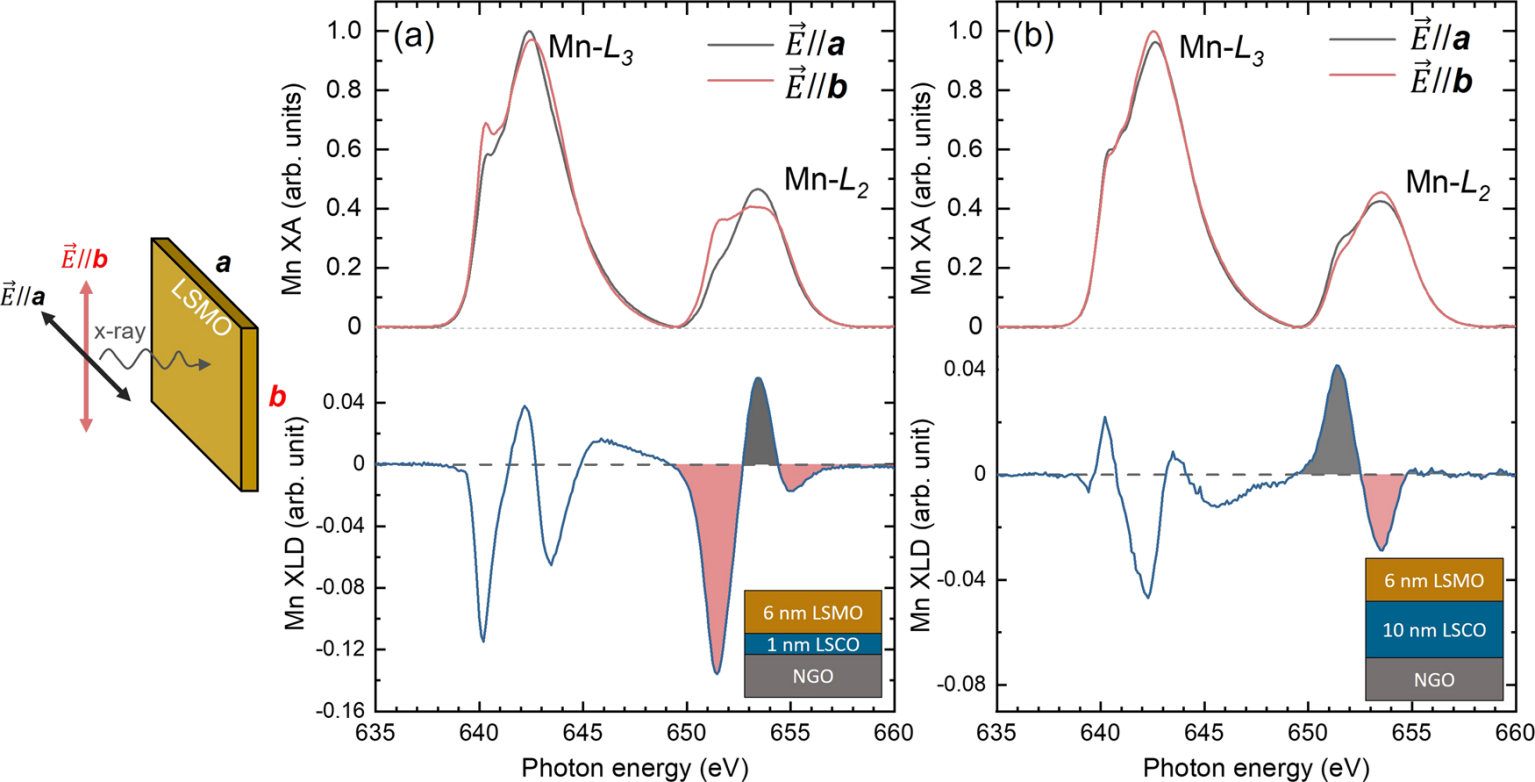
Schematic of the XA/XLD measurement geometry (left) for probing the 3*d*_*x*^2^–*y*^2^_ orbital occupancies along the ***a-*** and ***b-***directions, with X-rays perpendicular to the sample surface and *E⃗*//***a*** and *E⃗*//***b***, respectively. Mn *L*-edge XA (top) and XLD (bottom) obtained from (a) C1M6N and (b) C10M6N bilayers. The integrated area under the XLD spectra (*A*_XLD_) around the Mn-*L*_2_ peak is filled in gray and red for positive and negative XLD values, respectively.

Finally, XLD is an elemental-sensitive measurement technique for determining the preferred direction of 3*d* orbital occupancy in transition metal oxides which shows a direct correlation to MA.^26,57,58^ Here, we focus on probing the 3*d*_*x*^2^–*y*^2^_ occupancy because of its direct relationship with in-plane anisotropy. Mn-edge XA spectra were taken with X-rays perpendicular to the sample surface, while the polarization vectors have the geometry of *E⃗*//***a*** and *E⃗*//***b***, as shown schematically in Figure 6. It should be noted that XLD asymmetry may also arise from factors such as AFM ordering or ferromagnetism,^59^ but we can rule out the contribution from AFM ordering because of the bulk-like ferromagnetic behavior of the LSMO layer. The samples were demagnetized before the measurements, and no magnetic field was applied during the measurement. Figure 6a,b shows Mn *L*-edge XA/XLD spectra measured along the ***a***- and ***b***-directions of bilayers C1M6N and C10M6N, respectively. XLD was calculated as the difference between two XA spectra (*I*_XLD_ = *I*_***a***_*– I*_***b***_). The integrated area under XLD spectra (*A*_XLD_) around the Mn-*L*_2_ peak (from 649 to 660 eV) represents the preferred direction of Mn 3*d*_*x*^2^–*y*^2^_ orbital occupancy.^26^ The negative *A*_XLD_ from bilayer C1M6N reveals enhanced electron occupancy along the ***a***-direction that aligns well with the magnetically easy axis of the LSMO layer. In contrast, a positive *A*_XLD_ in bilayer C10M6N indicates favored electron occupancy along the ***b***-direction with the LSMO easy axis along the same direction. To examine the relationship between in-plane electron occupancy and the magnetic easy axis, Co-edge XA/XLD spectra were taken on bilayer C1M6N under the same conditions. The easy axis of the LSCO layer was along the ***a***-direction, while *A*_XLD_ around the Co-*L*_2_ peak (from 792 to 805 eV) was negative as shown in Figure S5. As a result, both Mn- and Co-edge XLD spectra reaffirm the direct correlation between the 3*d*_*x*^2^–*y*^2^_ orbital occupancy and in-plane MA.

Consistent with previous studies, the competition between BO_6_ octahedral rotation and strain has been identified as the primary factor that governs the MA of perovskite oxide heterostructures.^27,43^ In our study, no strain relaxation was observed with an increase in *t*_LSCO_ from 1 to 10 nm. The LSCO layer maintains its magnetic easy axis along the ***a***-direction, as the tensile strain state is smaller along this direction (0.57%) compared to that along the ***b***-direction (0.78%). Conversely, we found that the BO_6_ octahedra at the heterostructure interface play a significant role in determining the LSMO magnetic easy axis when *t*_LSCO_ ≤4 nm. In contrast to the rhombohedral structure in bulk LSCO and LSMO, LSCO/LSMO bilayers exhibit a monoclinic distortion, leading to biaxially anisotropic B–O bond lengths, thereby inducing symmetry breaking of the Mn 3*d*_*x*^2^–*y*^2^_ orbitals. The presence of Co^2+^ ions, which have a larger ionic radius, and oxygen vacancies can offer more degrees of freedom for octahedral rotation, thus eliminating the strain effect at the interface. Moreover, the FM exchange coupling between the soft LSCO and LSMO layers promotes the alignment of the LSMO easy axis to the LSCO easy axis, resulting in enhanced total magnetization of the LSCO layer and enhanced *H_c_* values of the LSMO layer. With further increase in *t*_LSCO_ beyond 4 nm, the concentration of Co^2+^ ions in the interface region decreases and the strain effect becomes the predominant factor influencing the MA of the bilayers. Consequently, the easy axis of the LSMO layer switches from IMA back to BMA, leading to a pronounced EB effect.

## 4 Conclusions

In conclusion, MA of a bulk-like LSMO thin film can be tuned by adjusting the thickness of the underlying LSCO layer in LSCO/LSMO bilayers grown under biaxial strain on NGO substrates. For *t*_LSCO_ ≤ 4 nm, no EB effect is observed along the ***a***-direction due to the strong interfacial magnetic coupling between the two layers that share the same in-plane magnetic easy axis and *H*_c_ values. However, along the ***b***-direction, a small negative exchange bias shift is observed because of the small *H*_c_ difference between the two layers. As *t*_LSCO_ increases beyond 4 nm, the EB effect appears due to the difference in MA of the two layers, causing the easy axis of the LSMO layer to rotate back to the [11̅0]_*o*_ direction as in bulk LSMO. With direct correlation to in-plane anisotropy, Mn-edge XLD spectra show different electron occupancy along the two in-plane directions with increasing *t*_LSCO_. Thus, the interfacial exchange coupling between the LSCO and LSMO layers facilitates the rotation of the LSMO easy axis, allowing for the control of the MA and EB between the two FM layers. Moreover, the ability to manipulate the MA of FM heterostructures, leading to thickness-dependent properties, makes magnetic perovskite oxide heterostructures promising candidates for next-generation spintronic and magnetic memory devices.

## Supporting Information Available

Structural characterization fitting results, coercivity derived from unbiased XMCD loops, and Co/Mn/O soft X-ray absorption spectroscopy data (PDF)
