# Supplementary material for: Tuning In-Plane Magnetic Anisotropy and Interfacial Exchange Coupling in Epitaxial La2/3Sr1/3CoO3/La2/3Sr1/3MnO3 Heterostructures
Source: ACS Appl Mater Interfaces. 2023 Nov 1;15(45):53086–95. doi: 10.1021/acsami.3c10376 (PMC10658449; doi:10.1021/acsami.3c10376)
Supplement: Supplementary file 1 — am3c10376_si_001.pdf [file am3c10376_si_001.pdf]

Supporting Information

Tuning In-plane Magnetic Anisotropy and Interfacial Exchange Coupling in Epitaxial $La_{2/3}Sr_{1/3}CoO_3/La_{2/3}Sr_{1/3}MnO_3$ Heterostructures

Mingzhen Feng,[1] Nolan Ahlm,[1] Dayne Y. Sasaki,[1] I-Ting Chiu,[2] Alpha T. N'Diaye,[3] Padraic Shafer,[3,†] Christoph Klewe,[3] Apurva Mehta,[4,†] Yayoi Takamura[1,]*

[1] Department of Materials Science and Engineering, University of California, Davis, Davis, California 95616, United States.
[2] Department of Chemical Engineering, University of California, Davis, Davis, California 95616, United States.
[3] Advanced Light Source, Lawrence Berkeley National Laboratory, Berkeley, California 94720, United States.
[4] Stanford Synchrotron Radiation Lightsource, SLAC National Accelerator Laboratory, Menlo Park, California 94025, United States.

Corresponding author email: ytakamura@ucdavis.edu

1. X-ray diffraction fitting results

Fitting parameters (thickness, density, and roughness of each layer) of RXRR curves for bilayer C4M6N are listed in the table below. A carbon capping layer was added to the fitting model due to the extended exposure to hard x-rays in air during the measurements. The total thickness of each layer is in a good agreement with expectations with an LSMO layer thickness of 5.76 nm and LSCO thickness of 4.23 nm.

Table SI: Fit parameters for RXRR spectra for bilayer C4M6N

Layer	Thickness (nm)	Roughness (nm)	Density (g/cm^3)
Carbon layer	1.84	0.97	2.32
LSMO surface	2.41	0.72	6.35
LSMO interface	3.35	0.41	6.02
LSCO interface	0.93	0.47	6.47
LSCO	3.32	0.62	6.72
NGO substrate	-	0.14	7.57

2. Coercivity (H_c) of LSCO and LSMO layers along *a*- and *b*-directions

H_c values from unbiased XMCD loops are plotted in Figure S1. A trend of increasing H_c (LSCO) as a function of t_{LSCO} is observed along both ***a***- and ***b***-directions. For thinner bilayers (C1M6N and C4M6N), H_c (LSCO) is nearly identical to H_c (LSMO) along the ***a***-direction, while exhibiting a slight difference along the ***b***-direction.

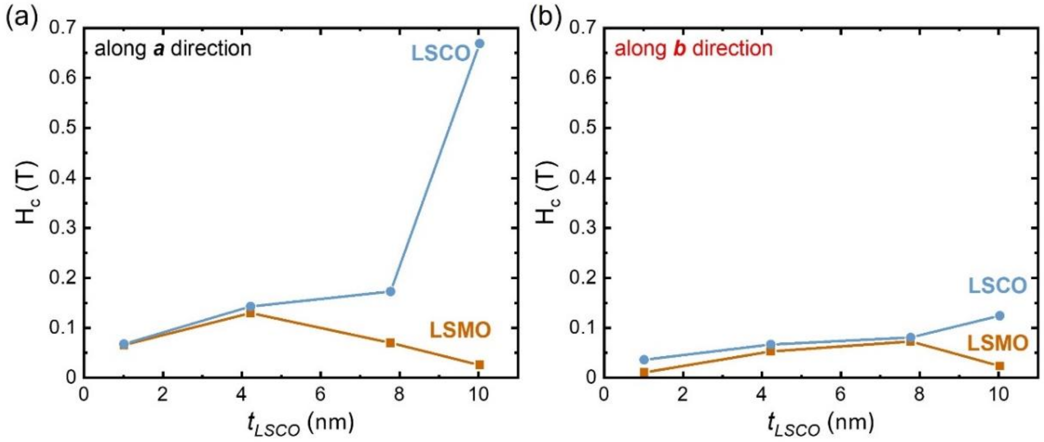

Figure S1. H_c as a function of t_{LSCO} derived from unbiased XMCD loops (a) along ***a*** ($[001]_o$) direction and (b) along ***b*** ($[1\bar{1}0]_o$) direction.

3. Co-edge XA/XMCD spectra with reference spectra

Figure S2 displays the Co *L*-edge XA/XMCD spectra of the bilayers, alongside reference spectra from a $CoFe_2O_4$ (Co^{2+}) thin film and a single-layer LSCO film containing mixed Co^{3+}/Co^{4+} ions. The XA/XMCD spectra have been normalized to the average XA peak at the L_3-edge, and for clarity, the curves have been vertically shifted. Analyzing the shape and peak positions of the spectra for bilayers C1M6N and C4M6N, it is evident that the Co^{2+} ions are predominantly present. However, for bilayers C8M6N and C10M6N, the Co-XA/XMCD curve closely resembles the reference spectra of the single-layer LSCO, indicating a prevalence of mixed Co^{3+}/Co^{4+} ions.

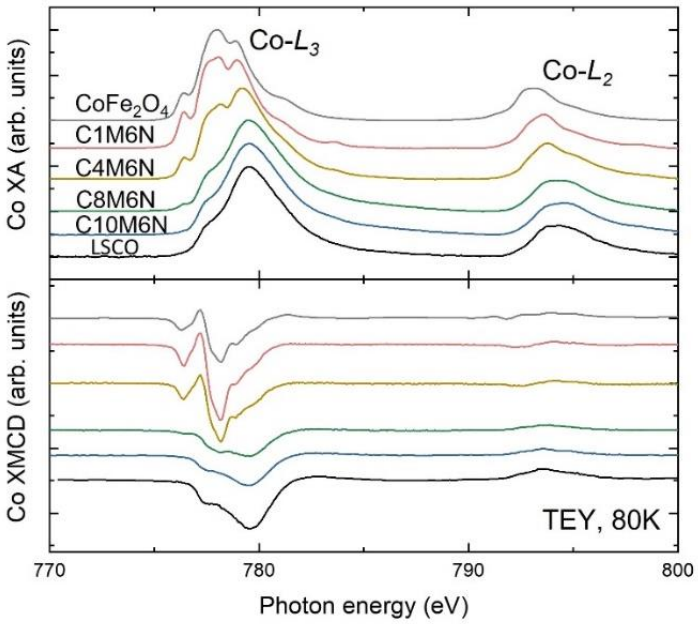

Figure S2. Co *L*-edge XA/XMCD spectra of LSCO/LSMO bilayers on NGO substrates taken in TEY mode at 80 K.

4. Mn-edge XA/XMCD spectra with reference spectra

Mn *L*-edge XA/XMCD spectra of bilayers and a single layer LSMO reference sample are plotted in Figure S3. The dashed line denotes the Mn-L_3 peak position from the single layer LSMO thin film. A small shift (~0.1 eV) of Mn-L_3 peak position of bilayers to higher energy can be observed, indicating slightly higher concentration of Mn^{4+} ions in LSCO/LSMO bilayers compare to a single layer LSMO thin film.

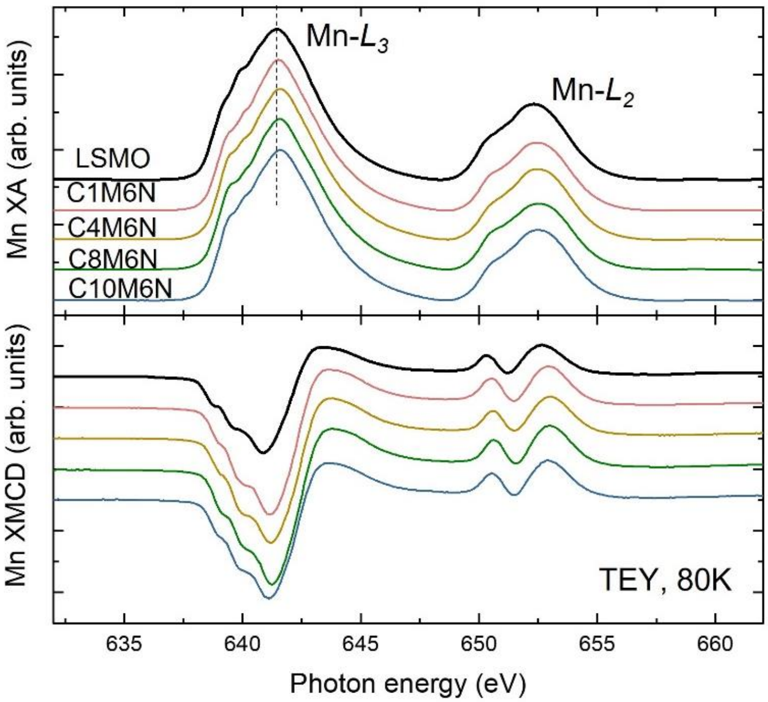

Figure S3. Mn *L*-edge XA/XMCD spectra of LSCO/LSMO bilayers on NGO substrates taken in TEY mode at 80 K. Reference spectrum from a LSMO single layer is also plotted. XA/XMCD spectra are normalized to the L_3-edge average XA peak and curves are vertically shifted for clarity.

5. O *K*-edge XA spectra

The O *K*-edge spectra of the bilayers were measured at 300 K, revealing evidence of more oxygen vacancies in the thinner bilayers. This result is indicated by a trend of decreasing intensity of peak A and increasing intensity of peak B as the LSCO thickness increases.

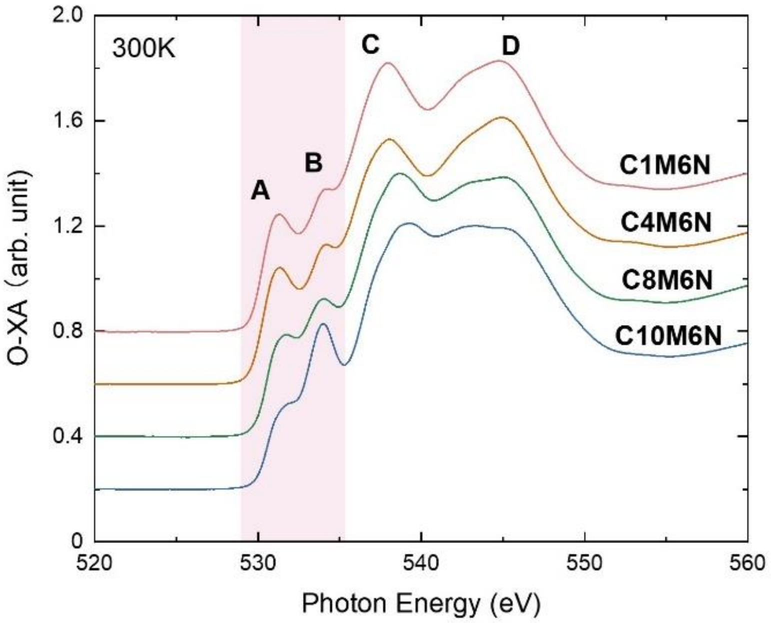

Figure S4. O *K*-edge spectra of LSCO/LSMO bilayers on NGO substrates measured at 300 K using TEY mode. Peak A: transition metal 3*d* unoccupied states; Peak B: transition metal 3*d* relevant absorption peak; Peak C: related to the band with La 5*d*/Sr 4*d*; Peak D: related to the band with Mn 4 *sp*.

6. Co-edge XA/XLD spectra on bilayer C1M6N

Figure S5 displays the Co *L*-edge XA/XLD spectra of bilayer C1M6N. The presence of negative integrated area, A_{XLD}, indicates that the easy axis of the LSCO layer aligns with the ***a***-direction, which is consistent with the alignment of the LSMO layer.

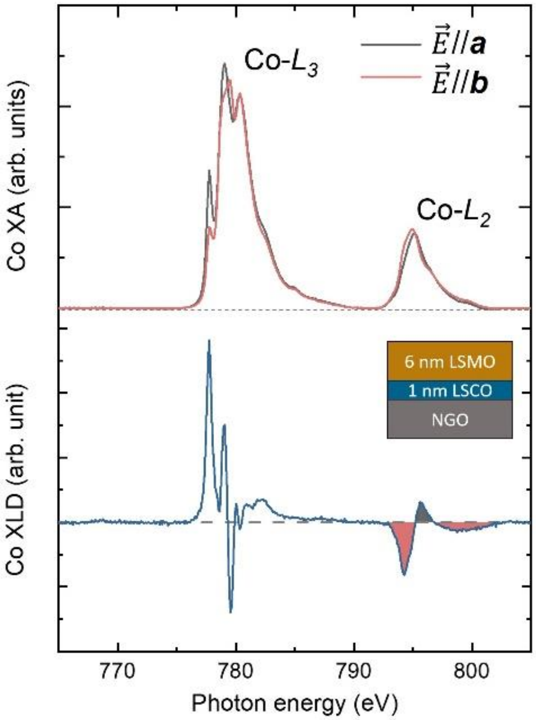

Figure S5. Co-edge XA/XLD spectra obtained from bilayer C1M6N taken in TEY mode at 80 K. X-rays are perpendicular to the sample surface with $\vec{E}//\boldsymbol{a}$ and $\vec{E}//\boldsymbol{b}$, respectively.
